# Gossypium Herbaceum—Some Remarks on Its Parturient Properties

**Published:** 1884-08-20

**Authors:** W. C. Bellamy

**Affiliations:** Atlanta, Ga.


### GOSSYPIUM HERBACEUM—SOME REMARKS ON ITS PARTURIENT PROPERTIES.

BY W. C. BELLAMY, M.D., ATLANTA, GA.

From a reprint from some foreign paper in the July No. of the Therapeutic Gazette, published in Detroit, Mich., I am glad to see that the parturient, if not the general emmenagogue properties of this article are at last attracting some attention in Europe, for as far back as 1863 I made the same experiments—and more, too—with even better results than are recorded in the article referred to, as having been made by Dr. Prochownich "*during the last four years.*"

In 1863, when the whole South was in a State of war and turmoil, and medicines contraband of war, it behooved us to develop such native remedies as our beloved Sunny South could supply us from her genial soil. Forced by the necessities of war, and following up the faint ray of light let in upon this subject by Doctors Shaw and Bouchelle, of Mississippi—the first I knew who ever recorded any observations on the subject—I commenced a series of experiments to ascertain the virtues, if any, of the eotton root.

My first experiment was in a case of labor, in company with an esteemed friend, Dr. Thos. J. Burke. The patient was found lying in an apparently indifferent, helpless condition; only an occasional pain, and very slight. An examination showed the uterus soft and flaccid, the os undilated and in an atonic condition, and the patient likely to become the victim of a long, tedious labor, if not of some subsequent trouble, also. No ergot to be had, unprepared with any apparatus for manufacture, and forced to use such a preparation as we could make "on the spur of the moment," I pulled up some of the roots from an adjoining field and made a simple decoction of about four ounces of the root to a quart of water boiled down to one half. The first dose, a small teacupful, showed its effects upon the uterus in twenty minutes by an increase in the force of the contractions. In half an hour the dose was repeated; the expulsive contractions became stronger and more frequent, the os began to dilate, and after two or three more doses, at longer intervals, the patient was easily and safely delivered, in four hours after the first dose, the placenta following readily. The woman, as soon as she could be made comfortable, fell into a sweet and refreshing slumber, and had no further trouble. Having previously borne several children, and although anticipating some difficulty, she afterward declared that she had "never had such an easy time before."

One remark of Dr. P.'s, in the article referred to, surprises me, since it does not coincide with my experience, nor appear to accord with reason. He says, "A fluid extract (made according to American Pharmacopœia, 1 gr. of extract equal to 1 gr. of root) may be given in one and two teaspoonful doses every half hour," but that "the intensity of action is not so great in the extract, and nearly double the quantity must be used." In my experiments, the reverse was the case. My greatest difficulty was that the bulk of the dose necessary to produce the desired effect was so great as to overload the stomach and nauseate the patient. This difficulty suggested the more concentrated form of a fluid extract, which was made for subsequent use, and was found to act admirably in doses of a dessert to a tablespoonful pro re nata, according to the judgment of the accoucheur.

One notable and fortunate fact developed itself in these experiments: that, in addition to its parturient effect, the medicine possessed marked anodyne properties, and while it increased both the force and frequency of the expulsive contractions, the actual pain attending them was diminished, and the patient obtained refreshing naps in the intervals between them.

During these experiments, in no single instance did any excessive hæmorrhage occur.

Such success having crowned these experiments, I reported them to several Journals, and, though my communications were extensively copied, beyond incorporating a short extract into the U. S. Dispensatory (from one of them in the Atlanta Medical and Surgical Journal), as to the best time to gather the root, the profession generally failed to manifest any interest in the matter. Hence, it is with pleasure that I see it, after the lapse of so many years, again attracting attention.

I received many letters on the subject, enquiring my mode of preparation, which I published many years ago; but, as it required more or less apparatus, and the process was more or less tedious when not prepared for it, it failed to come much into use. Of later years, all the manufacturing chemists of the country are making fluid extracts of this root; but they are, for the most part, unreliable, and their want of reliability is due, doubtless, to making it from old, dry root. Its virtues appear to be volatile, or, at least, to reside only in the *green root*, which, of course, cannot be had at the North, where most of them are made.

The extract, when properly made, has a beautiful, reddish-brown color, and, strongly, that unmistakable, peculiar odor so familiar to every Southerner, that in the darkest night will notify him by his sense of smell when he is near a gin house* or any pile of cotton seeds. Some few of the extracts on the market possess the *proper color*, but only very faintly the proper *odor*, while many, if not most of them, are of pale brandy color, with no odor except that of the alcohol, so that, in reality, very few of them possess the distinguishing characteristics that should lead one to place much reliance in their parturient or emmenagogue properties.

*A Southern "gin-house" is the building where the cotton is *ginned* or cleared of seeds and packed into bales for market, and a peculiar odor hangs around it, known only to Southerners or those who have seen one.

